# Metabolic and Microbial Modulation of the Large Intestine Ecosystem by Non-Absorbed Diet Phenolic Compounds: A Review

**DOI:** 10.3390/molecules200917429

**Published:** 2015-09-18

**Authors:** Juana I. Mosele, Alba Macià, Maria-José Motilva

**Affiliations:** Food Technology Department, Agrotecnio Research Center, University of Lleida, Av/Alcalde Rovira Roure 191, 25198-Lleida, Spain; E-Mails: juana.mosele@tecal.udl.cat (J.I.M.); albamacia@tecal.udl.cat (A.M.)

**Keywords:** colon metabolites, gut fermentation, microbiota, phenolic compounds

## Abstract

Phenolic compounds represent a diverse group of phytochemicals whose intake is associated with a wide spectrum of health benefits. As consequence of their low bioavailability, most of them reach the large intestine where, mediated by the action of local microbiota, a series of related microbial metabolites are accumulated. In the present review, gut microbial transformations of non-absorbed phenolic compounds are summarized. Several studies have reached a general consensus that unbalanced diets are associated with undesirable changes in gut metabolism that could be detrimental to intestinal health. In terms of explaining the possible effects of non-absorbed phenolic compounds, we have also gathered information regarded their influence on the local metabolism. For this purpose, a number of issues are discussed. Firstly, we consider the possible implications of phenolic compounds in the metabolism of colonic products, such as short chain fatty acids (SCFA), sterols (cholesterol and bile acids), and microbial products of non-absorbed proteins. Due to their being recognized as affective antioxidant and anti-inflammatory agents, the ability of phenolic compounds to counteract or suppress pro-oxidant and/or pro-inflammatory responses, triggered by bowel diseases, is also presented. The modulation of gut microbiota through dietetic maneuvers including phenolic compounds is also commented on. Although the available data seems to assume positive effects in terms of gut health protection, it is still insufficient for solid conclusions to be extracted, basically due to the lack of human trials to confirm the results obtained by the *in vitro* and animal studies. We consider that more emphasis should be focused on the study of phenolic compounds, particularly in their microbial metabolites, and their power to influence different aspects of gut health.

## 1. Introduction

Most of the beneficial health properties of fruit, vegetables, and whole grains have been attributed to bioactive non-nutritional chemical compounds commonly named phytochemicals, which include phenolic compounds. Plant phenols embrace a wide range of secondary metabolites that are synthesized from carbohydrates via the shikimate pathway, occurring as soluble conjugated (glycosides) and insoluble or bound forms [1]. Based on the extensive intake of these kinds of phytochemicals through the diet, a complex mixture of hundreds of phenolic compounds enters the gastrointestinal tract where they can be partially released and absorbed, or survive stomach and intestinal digestion and reach the colon until excretion via feces. Several studies have reported that an important part of the ingested phenolic compounds reaches the large intestine where it undergoes a series of microbial transformations that leads to the generation of related metabolites [2,3,4].

On occasion, gut microbiota has been defined as a biological reactor since it possesses powerful metabolic functions which include the transformation of many compounds that reach the colon. This activity is possible through the capacity of microorganisms to produce a huge and varied range of enzymes. In the particular case of phenolic compounds, their intestinal transformations include several steps. First, aglycones must be released to the media. For this, different classes of enzymes are needed to deconjugate the specific moiety associated to the molecule or, in the case of polymeric forms, to break phenolic polymers into individual monomers. Phenolic compounds are also strongly linked to some components of the food matrix, and this interaction is also disrupted by microbiota [1]. The released aglycones undergo subsequent microbial transformations which may include ring fission, α or β-oxidation, dehydrogenation, dehydroxylation, and demethylation, and these result in the generation of simpler related compounds.

**Figure 1:**
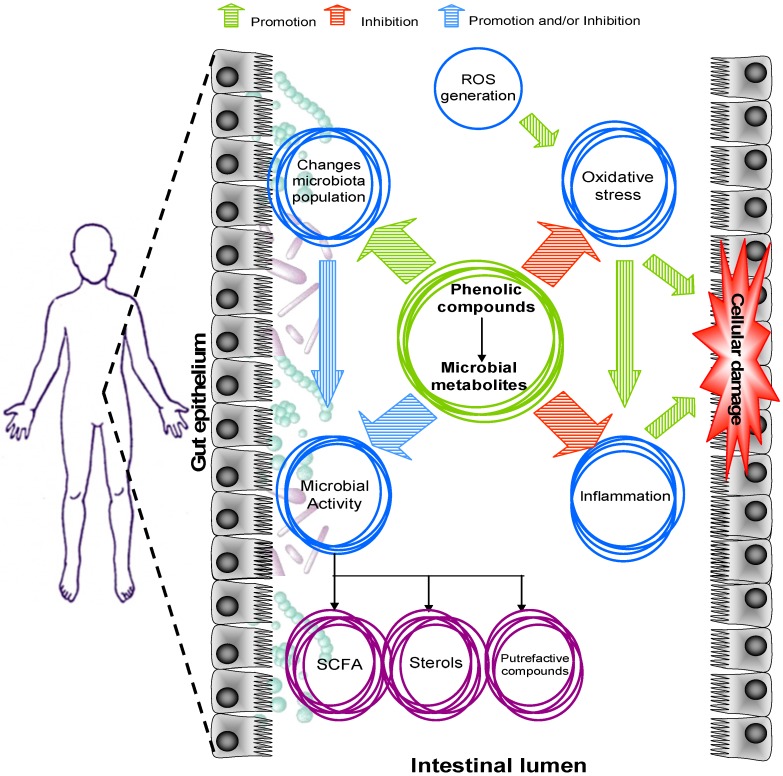
Overview of the possible implications of phenolic compounds at the gut level.

An overview of the bioactivity that is probably carried out by phenolic compounds in the large intestine is represented in Figure 1. The passage of digesta through the small intestine is estimated to take around 2–4 h, but the transit time increases considerably in the large intestine, extending to as long as 24 h or more [4,5]. This time is probably long enough to accumulate substantial amounts of phenolic compounds and induce metabolic and microbial changes in the gut lumen. Phenolic compounds are recognized antioxidants and anti-inflammatory agents which can protect intestinal cells from pro-oxidant and inflammatory injuries [6,7,8]. The presence of luminal phenolic compounds could also impact the metabolic profile, enhancing or inhibiting the generation of fermentation products derived from endogenous and dietary compounds. In turn, changes in the metabolic profile of the gut are sometimes associated with the modification of the shape of the intestinal inhabitants. In fact, some authors have associated the phenolic compounds–gut microbiota interaction with a presumable modulation effect, which could prevent or, indeed, restore microbiota alterations observed in disease [9,10].

The aim of this manuscript was to consult the information available from *in vitro* and *in vivo* studies to review the transformations that the non-digested phenolic compounds undergo when they reach the colon and the possible influence they have on the transformation of other intestinal metabolites, such as short chain fatty acids (SCFAs), sterols, and microbial products of non-absorbed proteins. In addition, their possible implications in metabolic and microbial changes as well as in antioxidant and anti-inflammatory effects at the gut level were also considered.

## 2. Microbial Phenolic Metabolites

Phenolic compounds are commonly classified into two main groups, flavonoids and non-flavonoids. In the next section we will describe the catabolism pathways and the phenolic metabolites formed related with the chemical structures of flavonoids and non-flavonoids.

### 2.1. Flavonoids

Flavonoids possess an aglycone skeleton of two flavonoid rings (A- and B-rings) connected by a heterocyclic C-ring. In foods, except for flavan-3-ols, flavonoids are normally presented as glycosides or organic acid conjugates.

**Figure 2:**
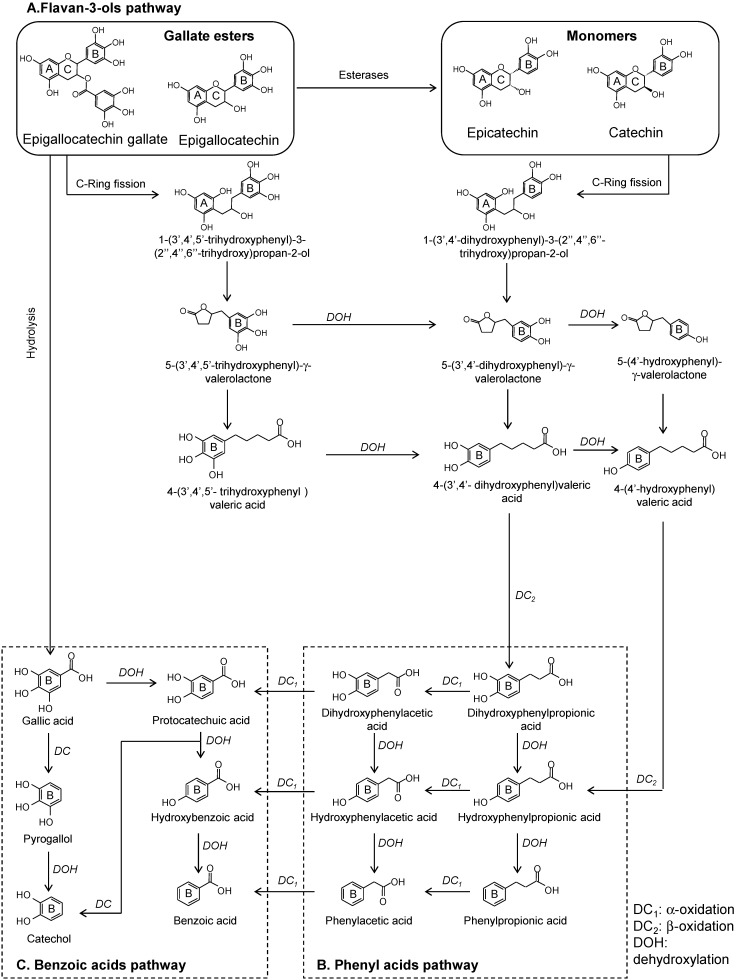
Proposed colonic pathways of monomeric flavan-3-ols and their corresponding gallate esters.

The sub-class of flavan-3-ols includes the diastereomers catechin and epicatechin, and their corresponding gallate esters, epigallocatechin and epigallocatechin gallate. In foods, flavan-3-ols are present as monomers (catechin and epicatechin) or proanthocyanidins (variable attached monomers). To study the microbial modifications of flavan-3-ols, pure standards as well as grapes, tea, cocoa, berries, red wine, and their extracts have been used. The colonic catabolism of proanthocyanins undergoes a first degradation step resulting in the release of monomer structures with the subsequent hydrolysis of gallic acid from the galloylated forms [11,12,13,14]. Based on the literature, we propose the colonic pathways of monomeric flavan-3-ols and their corresponding gallate esters (Figure 2). The early appearance of simple phenolics (like catechol) suggests further degradation of gallic acid generated by hydrolysis of epigallocatechin gallate in the first steps of the colonic catabolism [11,12,14,15]. Similarly, the initial C-ring fission of flavan-3-ols (Figure 2A) produces the corresponding diphenylpropan-2-ol. Then, this is further converted into 5-(3′,4′,5′)-tri- and 5-(3′,4′)-dihydroxyphenyl-γ-valerolactone in the cases of gallate esters and monomers, respectively [11,14,16]. The subsequent catabolism of the tri- and dihydroxyphenyl-γ-valerolactones originates different hydroxylated forms of phenylvaleric acid [11,12,13,14,15,17]. Neither trihydroxyphenylvaleric acid nor trihydroxyphenylpropionic acid have been described as microbial metabolites of gallate esters in *in vitro* fermentations. However, trihydroxyphenylvaleric acid was identified in human plasma [16]. Phenyl-γ-valerolactones and phenylvaleric acids have been described as exclusive microbial metabolites of flavan-3-ols. Their progressive microbial transformation releases different hydroxylated forms of phenyl (Figure 2B) and benzoic acids (Figure 2C), whose profile and abundance seem to depend on the particular metabolic activity of each individual microbiota and the composition of flavan-3-ols in the substrate [11,12,13,14,15,17]. Furthermore, other minor catabolites, such as hippuric acid, *p*-coumaric acid, and vanillic acid, homovanillic acid, homovanillyl alcohol, and 3-*O*-methylgallic acid have been associated with the *in vivo* colon metabolism of flavan-3-ols [11,13,16,18].

**Figure 3:**
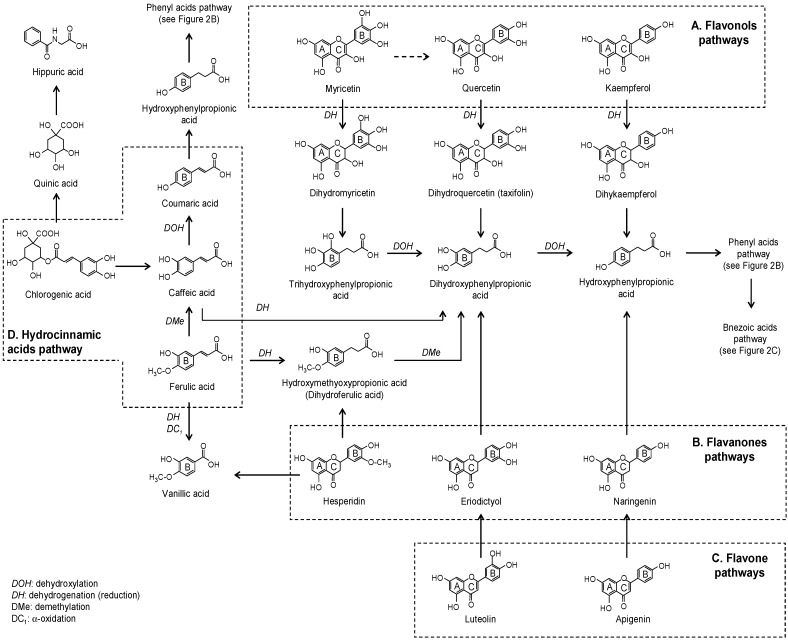
Proposed colonic pathways of flavonols, flavanones, flavones, and hydrocinnamic acids.

Flavonols are the most common phenolic compounds in foods such as red wine, apples, onions, beer, spices, herbs, berries, and cocoa, with quercetin, kaempferol, and myricetin being the most studied. During the first steps of the colonic catabolism of the aglycone forms of quercetin and kaempferol, the initial metabolites formed are dihydroquercetin (taxifolin) and dihydrokaempferol, respectively, which are further metabolized to di- and hydroxyphenylpropionic acid, respectively (Figure 3A) [19,20,21,22,23]. These metabolites then enter the catabolic route of phenyl acids (Figure 2B) and benzoic acids (Figure 2C) to generate minor related compounds [19,22,23]. Following the intake of radiolabeled quercetin-4′-*O*-glucoside in rats, high concentrations of hippuric acid were found in the jejunum/ileum, urine, and plasma [22]. Furthermore, 3′,4′,5′-trihydroxyphenylacetic acid and 3′,5′-dihydroxyphenylacetic acid were the main microbial metabolites detected in rat feces after the administration of myricetin [24], probably derived from the intermediate dyhydromyricetin. However, the generation of quercetin from myricetin should not be discarded [25].

The flavanones hesperetin and naringenin are found in larger concentration in oranges, whereas eriodictyol is a flavanone commonly presented in aromatic herbs and nuts. Hydroxyphenylpropionic and phenylpropionic acids have been described as major fermentation products of eriodictyol and naringenin [23,26,27]. In addition, phloroglucynol was also detected in the fermentation vessels of naringenin [23] and eriodyctiol [28] which could be formed from the A-ring. In the case of hesperidin, 3-(3-hydroxy-4-methoxyphenyl)propionic acid (dihydroisoferulic acid) and different hydroxylated forms of phenylpropionic acid have been reported as products of it colonic catabolism [27] (Figure 3B).

Flavones, like apigenin and luteolin and their glycosides, are normally found in beer, olive oil, aromatic herbs, and nuts. In the same way as with the other flavonoid sub-classes, deglycosylation is the first microbial action. Apigenin aglycone is transformed into naringenin, whose microbial catabolism produces phenylpropionic acids [21,29] (Figure 3C). Luteolin undergoes an initial isomerization to form eriodictyol as an intermediate metabolite which is transformed to 3-(3′,4′-dihydroxypheyl)propionic acid [21,30] (Figure 3C). In coincidence with *in vitro* data, 3-(4′-hydroxyphenyl)propionic acid was the main metabolite detected in urine after the administration of apigenin-7-*O*-glucoside to human microbiota-associated rats [29].

**Figure 4:**
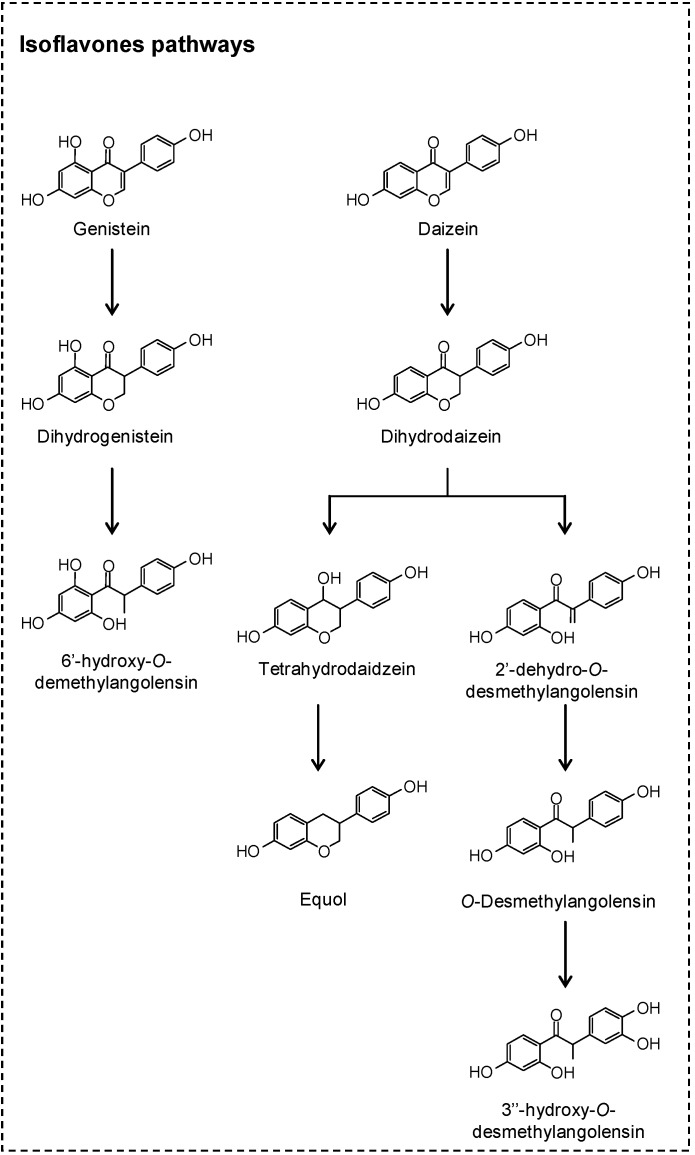
Proposed colonic pathways of isoflavones.

Genistein and daidzein are the most representative isoflavones and are normally present in their glycoside forms in soy products. The results of *in vitro* incubations of genistein, together with experiments where gnotobiotic rats were inoculated with isolated human equol-forming bacteria, have shown that, during the first steps of colonic catabolism, genistein, aglycone generated dihydrogenistein (Figure 4) which is partly converted into 5-hydroxy-equol [31,32]. After *in vitro* fermentation of genistein, Coldham *et al.* [33] observed that dihydrogenistein was metabolized into 6′-hydroxy-*O*-demethylangolensin which, in turn, was transformed into 2-(4-hydroxyphenyl)propionic acid and phloroglucinol. On the other hand, daidzein aglycone can be reduced to dihydrodaidzein and then converted into equol or *O*-desmethylangolensin [31,32,34] (Figure 4). Although more than one bacteria has been described as an isoflavone-converter [31,32], not all humans have the capacity to convert genistein and daidzein into their respective microbial metabolites [35], which could indicate that isoflavone transformer bacteria are not common among human intestinal flora.

**Figure 5:**
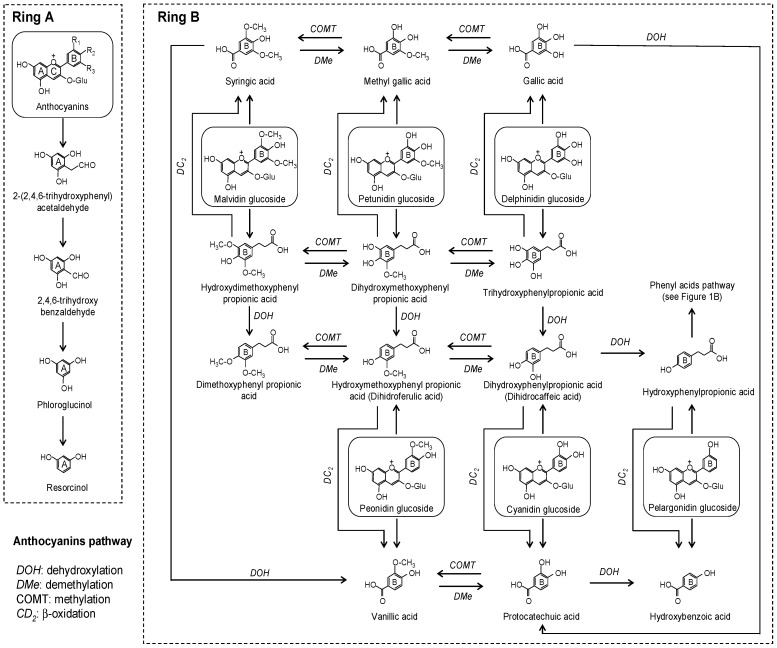
Proposed colonic pathways of anthocyanins.

Anthocyanins are widely dispersed throughout the plant kingdom, being responsible for red, blue, and purple colors. Red wine, grapes, berries, and pomegranate are examples of anthocyanin-rich products. After microbial deglycosylation, ring fission of the aglycone releases two parts, one from the A-ring and the second one from the B-ring, which then undergo simultaneous catabolism [36,37]. Regarding the fission of the B-ring, both phenyl acids and benzoic acids have been reported as the microbial metabolites of anthocyanins (Figure 5). Gonzalez-Barrio *et al.* [37] proposed a complete colonic pathway for cyanidin including different alternative conversion pathways for the B-ring, and protocatechuic acid (benzoic acid) and 3-(3′,4′)-dihydroxyphenylpropionic acid (phenyl acid) were proposed as initial metabolites. The subsequent microbial metabolism of these initial products generates simpler compounds. Regarding benzoic acids, hydroxybenzoic acid has been reported to be a microbial metabolite of pelargonidin glucoside, protocatechuic acid of cyanidin glucoside, vanillic acid of peonidin glucoside, syringic acid of malvidin glucoside, methyl gallic acid of petunidin glucoside, and gallic acid of delphinidin glucoside [36,37,38,39,40]. Considering the results obtained from previous studies, the generation of benzoic acids prevails over that of phenyl acids (Figure 2B,C and Figure 5).

Apart from B-ring fission, other microbial metabolites could also be generated from A-ring fission. The fission of ring A of anthocyanins could generate trihydroxybenzaldehyde [39,40], which could further be converted to phloroglucinol [37]. Due to the lack of complete information regarding the description of the colonic fate of some anthocyanins, we tentatively propose alternative pathways in Figure 5. Obviously, the acceptance or rejection of these must be confirmed by future studies. Along with some of the metabolites described in *in vitro* studies, hippuric acid was also detected in human urine after the ingestion of raspberries [37].

### 2.2. No Flavonoids

Phenolic acids, such as hydroxycinnamic and hydroxybenzoic acids, are present in numerous plant products. In plant tissues, phenolic acids form ether linkages with lignin through their hydroxyl groups in the aromatic ring and ester linkages with structural carbohydrates and proteins through their carboxylic group [1]. These bound phenolics survive stomach and intestinal digestion and reach the colon since, being the substrate to colon microbiota, cell wall fibrous materials are difficult to digest.

Hydroxycinnamic acids are widely distributed in nature, with coffee, whole cereals, dried drupes, wine, berries, spices, and aromatic herbs being the richest sources. Caffeic, ferulic, and *p*-coumaric acids, as well as their tartaric and quinate esters, such as chlorogenic acid (quinate ester of caffeic acid), are included in this group, and their microbial degradation steps are proposed in Figure 3D. Chlorogenic acid underwent dihydroxylation, dehydrogenation, or ester hydrolysis as a first microbial transformation in simulated conditions [41]. *In vitro* fermentation of freeze-dried coffee confirmed the initial hydrolysis of chlorogenic acid into caffeic acid which later suffers degradation, with di- and mono-hydroxylated phenylpropionic acids the main metabolites detected [23,42]. Other metabolites, such as *m*-coumaric and hippuric acids, were detected in the urine of rats after the administration of chlorogenic acid [43]. As an intermediate product of chlorogenic acid, caffeic acid degradation was expected to produce a similar metabolic profile [43] (Figure 3D). Dihydroferulic acid (3-(3-methoxy-4-hydroxyphenyl)propionic acid) [27,39] and 4-vinylguaiacol [44] together with minor degradation compounds have been described after *in vitro* fermentation of ferulic acid.

On the other hand, hydroxybenzoic acids are made up of gallic acid and ellagic acid. Gallic acid is also part of the hydrolysable tannins’ and flavan-3-ols’ molecular structure. Its microbial transformation is proposed in Figure 2B, whereas the colonic fate of ellagic acid is explained in the following section.

**Figure 6:**
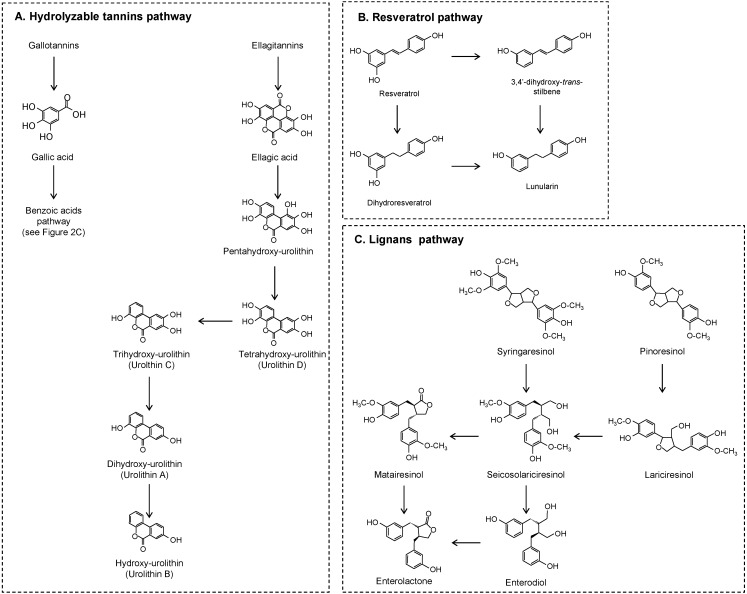
Proposed colonic pathways of hydrolysable tannins, resveratrol, and lignans.

Ellagitannins are the main group of hydrolysable tannins. Particularly high concentrations of ellagitannins are found in muscardine grapes, pomegranates, and some berries and nuts. Intestinal breakdown of ellagitannins into ellagic acid was observed *in vitro* [2,37,45] and *in vivo* [46]. Ellagic acid is further metabolized by local bacteria, giving pentahydroxy-urolithins as the first metabolite which is successively dehydroxylated to tetra-, tri-, di-, and mono-hydroxy-urolithins, [2,37,45,46,47], as proposed in Figure 6A. An important person-to-person variability in the profile and amounts of urolithins has been observed with major urolithin A (dihydroxy-urolithin) or urolithin B (hydroxy-urolithin) producers, and others are incapable of producing any class of urolithins [2,3,37,45,47]. *Gordonibacter urolithinfaciens* and *Gordonibacter pamelaeae* were described as responsible for the transformation of ellagic acid into penta-, tetra-, and trihydroxyurolithin [48] and its absence could be associated with the inability of some individuals to produce urolithins.

Resveratrol (3,5,4′-trihydroxy-*trans*-stilbene) is the most common stilbene, present in grapes and wine, and it has been extensively studied. Regarding the *in vitro* experiments, dihydroresveratrol, 3,4′-dihydoxy-*trans*-stilbene, and lunularin (3,4′-dihydroxy-bibenzyl) were described as microbial derivatives [49,50] (Figure 6B). Bode *et al.* [49] concluded that three colonic pathways of resveratrol can be distinguished in terms of the quantity of end products as a lunularin producer, dihydroresveratrol/lunularin producer, or dihydroresveratrol producer. Whereas dihydroresveratrol and lunularin were considered the main end products, the low amounts of 3,4′-dihydroxy-*trans*-stilbene detected seem to indicate that this product is intermediate or marginally produced by gut microbiota. The urine metabolic profile after an acute dose of *trans*-resveratrol confirmed the results found under *in vitro* fermentation [49,50].

Syringaresinol, pinoresinol, lariciresinol, secoisolariciresinol, and matairesinol are the most common lignans found in plant products. Besides being present in foods, secoisolariciresinol is a microbial degradation product of syringaresinol and also of lariciresinol, which, in turn, is derived from the microbial fermentation of pinoresinol [51,52] (Figure 6C). The final microbial products of secoisolariciresinol, with several intermediates, are enterodiol and its oxidized product, enterolactone [53,54]. Secoisolariciresinol can also be converted to matairesinol, from which only enterolactone is obtained from microbial catabolism [52].

**Figure 7:**
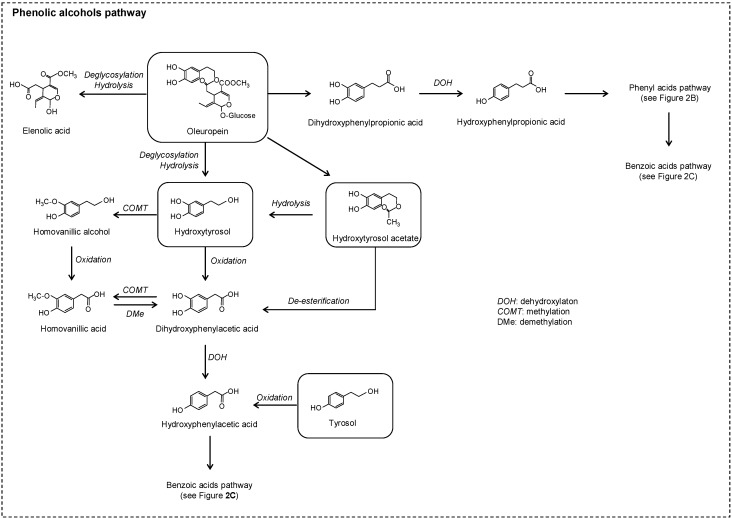
Proposed colonic pathways of oleuropein.

The phenolic alcohols, tyrosol, hydroxytyrosol and its precursors, oleuropein, and hydroxytyrosol acetate are the most representative compounds typically found in olives and virgin olive oil. Few studies have focused on the colonic pathway of these phenolic compounds. Oleuropein was transformed *in vitro* into its aglycone, elenolic acid, hydroxytyrosol, and hydroxytyrosol acetate [55,56] (Figure 7). Individual fermentation of tyrosol and hydroxytyrosol confirmed the low microbial metabolism of these compounds and their relative stability under *in vitro* colonic conditions [55]. An *in vivo* experiment following the administration of oleuropein to rats detected other related metabolites such as homovanillic acid [57].

Considering the complex colonic pathways, it is possible to conclude that the first microbial transformation of phenolic compounds leads to the accumulation of initial metabolites that share characteristics from the original compound (particularly conserving their functional groups) and are considered markers of an early fermentation stage. The continuous exposure of the initial metabolites to microbiota leads to the accumulation of final catabolites, which can be common to different phenolic sub-groups. An elevated accumulation of some microbial metabolites would be indicative of the main microbial pathways probably common to several individuals. On the contrary, those metabolites present in minor concentrations or in a smaller proportion of individuals could be related to a specific microbiota composition capable of activating secondary metabolic pathways.

Different profiles of metabolites observed after *in vivo* interventions suggest hepatic and renal metabolism prior to excretion via urine. Hippuric acid was the common phenolic metabolite detected in the largest amounts in plasma and urine after the intake of varied phenolic sources. The origin of hippuric acid can be through the microbial transformation of the quinic acid moiety or the hepatic metabolism of benzoic acids [58,59]. Nevertheless, the concentration of hippuric acid in urine decreased considerably after the antibiotic treatment of rats [60] and in the urine of ileostomy [11], suggesting an important contribution by the gut microbiota.

*Box 1*. ***Models Concerning the Study of Phenolic Microbial Metabolites***

*In vitro* and *in vivo* studies have been essential for building the colonic pathways of phenolic compounds. Basically, *in vitro* studies consist of anaerobic incubations where phenolic compounds represent the microbial substrate and bacteria cultures, human or animal feces, the microbial inoculum. The phenolic substrate used in these studies may include pure standards [11,19,20,26,30,38,49,55], isolated compounds [36,38,45], phenolic extracts [12,15,38,39,45], and food or pre-digested food [2,37,42,57]. *In vitro* fermentation models have some questionable points, mainly their limited representativeness of *in vivo* conditions. For example, *in vitro* incubations do not include enterohepatic recirculation, colonocyte absorption, mucosa-associated microbiota, and changes in physiological conditions during the transit time. The latter point has been partly overcome with the development of modern controlled batch cultures called “Simulator of Human Intestinal Microbial Ecosystem” (SHIME). The sample circulates through different reactors that represent the ascending, transversal, and descending colon in which pH and temperature are continuously controlled and the growth of microbiota is in line with the environmental conditions and substrate availability [12,17,61]. Despite the mentioned limitations, *in vitro* studies provide valuable information regarding the phenolic metabolites generated by gut microbiota. Fermentations are usually performed for 24–48 h during which sub-samples are collected at different time-points. Time-course metabolite generation allows colon metabolic pathways that probably take place under *in vivo* conditions to be defined. As microbial metabolites can be absorbed by colonocytes, their *in vitro* identification provides a useful tool for identifying these phenolic metabolites in plasma and/or urine in the human bioavailability or dietary intervention studies.

*In vivo* studies are ideal complements to the *in vitro* ones. Nevertheless, complex ethical issues, concerning the study of the phenomena occurring during human digestion, limit *in situ* observations and sample collection. In general, phenolic compounds are usually analyzed in fractionated blood (plasma, serum…) and urine to determine which compounds are absorbed and their absorption and excretion kinetics. In general, phenolic compounds are quickly absorbed, reaching the C_max_ in plasma between 0.5 and four hours after the intake, corresponding to the stomach and/or small intestine absorption [16,18,37,62], whereas the colonic metabolites appear in plasma later, indicating their gradual colonic biotransformation and, in some cases, showing a second increase in the plasma concentration after 4 h [16,18].

*In vitro* and *in vivo* studies have contributed enough so far to confirm the capacity of gut microbiota to metabolize phenolic compounds (Box 1). The degradation pathway routes involve several reactions in which the side chain groups and the heterocyclic C-ring of flavonoids are more likely to be used by microorganisms, while the benzoic ring remains intact. Depletion of phenolic compounds along with the increase in bacteria growth observed in *in vitro* fermentations could be considered good evidence to believe that these phytochemicals are used as carbon and energy sources. However, there are other reasons that can explain the utilization of phenolic compounds by gut microbiota. As xenobiotics, they may be degraded to reduce the toxicity that these compounds may have on resident bacteria.

## 3. Impact of Diet Phenolic Compounds on Gut Microbial Fermentation Metabolites

### 3.1. Short Chain Fatty Acids (SCFAs)

SCFAs are saturated aliphatic organic acids consisting of one to six carbons, of which acetic (C2), propionic (C3), and butyric (C4) acids are the most abundant. SCFAs production mainly occurs in the proximal part of the colon, where the availability of substrates is most abundant. The majority of SCFAs (up to 95%) are rapidly absorbed by the colonocytes, leading to decreasing concentrations from the proximal to distal colon. Only a minor fraction of the SCFAs (about 5%) is excreted in the feces [63]. Isobutyric, isovaleric, and valeric acids, also called branched chain fatty acids (BCFAs), constitute the remaining 5% and are derived from the microbial fermentation of branched chain amino acids [64,65]. A disruption in the concentration of SCFAs, especially due to an increase in the proportion of BCFAs, could be a possible signal of loss of intestinal homeostasis [66,67].

Acetic acid, propionic acid, and particularly butyric acid have been extensively studied due to their involvement in the maintenance of correct body functions [68,69]. Butyric acid is considered essential for maintaining the colon cellular function since it is the main energy source for colonocytes [70]. Chemopreventive properties have also been described for butyric acid due to its capacity to prevent the formation of malignant cells and to induce apoptosis in colonic cancer cells [71]. In fact, lower amounts of SCFAs [72] and butyric acid-producing bacteria [73] were found in the feces of colorectal patients than in those of healthy subjects. Contrarily, elevated amounts of BCFAs are presumed to be prejudicial to colonic health [74,75] and high concentrations have been observed in inflammatory bowel diseases (IBDs) [66] and obesity [67].

*In vitro* fermentation of pure phenolic compounds enables the extent to which phenolic compounds and gut microbiota are exclusively involved in SCFAs production to be seen. An improvement in the total production of SCFAs was observed after the *in vitro* incubation of chlorogenic acid, rutin, caffeic acid, quercetin [76], and olive oil polyphenols [77]. To the contrary, isolated proanthocyanidins [78] and punicalagins [45] suppressed the *in vitro* generation of SCFAs. The increments observed leave interpretations open to the thought that phenolic compounds could be transformed into SCFAs. However, this alternative is practically discarded since the ring cleavage of the aromatic ring of phenolic compounds was not observed under anaerobic conditions [79]. Rather than direct production, the increase in SCFAs observed after *in vitro* fermentation of pure phenolic standards is probably related to a higher fermentation rate of the released glycoside moieties and/or of the remaining carbohydrates in the culture media or the feces.

Phenolic-rich extracts have increased in interest since they can provide added value as food ingredients and can also be used in nutraceutical applications. Their involvement in SCFAs generation has shown mixed results. In two experiments, black tea extract, red wine-grape juice extract [17], and soy germ powder [61] were studied in a SHIME *in vitro* system. The tea extract and soy germ powder increased the concentration of acetic and propionic acids, whereas the soy germ and the mix extract only increased the concentration of propionic acid. In parallel, a slight decrease in the amount of butyric acid was observed after continuous feeding of black tea and red wine-grape juice extracts. A rise in the production of the three main SCFAs was observed after incubation of pomegranate extract [45]. Higher recoveries of urolithins in the batch cultures of pomegranate extract compared with that of punicalagins suggest major microbial activity and a possible involvement of phenolic microbial metabolites. Nevertheless, rats fed with raspberry seed extract produced more SCFAs and minor urolithins than rats that received strawberry seed extract [80]. Thus, the results of different studies are contradictory and phenolic compounds with similar chemical characteristics showed different behavior.

Because the later data was obtained under *in vitro* conditions, it risky to extrapolate the same effects to *in vivo* conditions when extracts or phenolic compounds are ingested through the diet. Heterogeneous data have also been obtained from animal and human studies after exposure to a phenolic-rich diet. The concentration of acetic acid increased in the feces of pigs after two weeks of receiving a diet enriched with 0.2% of green tea phenolic compounds [81]. In healthy humans, the inclusion of a red wine-grape juice extract, but not grape juice extract, over four weeks, reduced the concentration of isobutyric acid [82]. Furthermore, a decrease in the production of SCFAs in rats was noticed after the administration of an aqueous passion fruit leaf extract [83]. No changes in the production of SCFAs were observed after the inclusion of trans-resveratrol or quercetin (alone or in combination) in rats fed with a high-fat sucrose diet for six weeks [84] or in healthy humans after the intake of pomegranate juice for four weeks [85].

The role of non-digestible carbohydrates (dietary fiber) in the production of SCFAs is unquestionable. Based on its chemical and physical properties, dietary fiber is normally classified into soluble and insoluble. Pectin, oligosaccharides (fructooligosaccharides, FOS), and inulin are considered soluble fiber. The fermentability of soluble fiber is high due to its capacity for water solubilization, which allows the action of microbial enzymes. Insoluble fiber corresponds basically to cellulose, some hemicelluloses, lignin, and arabinoxylans, which are also fermented, but at a much lower rate than soluble fiber [86]. Many authors have postulated that the production of SCFAs may be enhanced when dietary fiber is administered in combination with phenolic compounds. However, this claim remains inconclusive.

Replacement of part of the cellulose (maize starch) by strawberry and raspberry defatted seed extracts rich in ellagitannins enhanced the total SCFAs production and the proportion of butyric acid in rats after four weeks [80]. However, the results of the latter study were not only attributed to the phenolic compounds since their effectiveness was more marked in the raspberry extracts, coinciding with a major content of soluble fiber. In a similar experimental design, equivalent concentrations of dietary fiber were included in the diets of rats through soluble and insoluble fractions of bilberries, blackcurrants, and raspberries [87]. Rats fed with the soluble fraction of bilberries showed the highest caecal formation of SCFAs and fiber fermentability in coincidence with higher anthocyanin intake. Moreover, the addition of apple-pomace extract, in combination with FOS and cellulose, for four weeks [88], or lyophilized apple together with apple pectin [89], increased the proportion of butyric acid in rats compared with when fiber was consumed alone, suggesting an additional benefit that could be attributed to the presence of phenolic compounds in the extract.

In some cases, the combination of fiber with phenolic compounds seems not to alter the gut metabolism of SCFAs. For example, apple fiber rich in phenolic compounds did not produce any change in the generation of SCFAs, either in combination with apple pectin under *in vitro* conditions [71] or together with boysenberry juice after four weeks of human consumption [90]. In line with the previous studies, rats fed with apple-pomace extract rich in proanthocyanins showed no difference in the production of SCFAs compared with rats fed with apple-pomace extract deprived of phenolic compounds [91], and similar results were observed after combination of grape extract and inulin in the diet of rats [92]. Inhibitory effects in the production of SCFAs were observed when isolated apple polysaccharides were fermented *in vitro* together with purified apple phenolic extract rich in proanthocyanins [79]. Similarly, the suppression of SCFAs was also noticed in rats after four weeks of a combination of wheat and oat fiber with blackcurrant and chokeberry extracts (both rich in proanthocyanidins) [93] and low or high ellagitannin-rich strawberry extract in combination with FOS [94]. The conversion of SCFAs from starch was also suppressed by the addition of grape-seed extract to pig fecal incubations [95].

Since many factors such as the abundance and activity of specialized carbohydrate-fermented bacteria and enzyme expression and activity, as well as substrate availability, are involved in SCFAs synthesis, phenolic compounds could interact with one or more of these factors. Depending on the type and amount of the phenolic compounds ingested, they reach the colon in different proportions and degrees of polymerization [4,62]. In accordance with this, they could promote or inhibit the growth of carbohydrate-fermented bacteria [76,96]. Bacterial enzymatic expression and activity is crucial for the release of monomeric compounds from polysaccharides to form SCFAs. In addition, different works suggest that polyphenols might modulate the activity of glyosidic enzymes differently [80,92,93].

In plant foods, phenolic compounds are naturally associated with dietary fiber. Thus, the physiological effects of fiber may depend not only on its chemical and physical properties, but also on its particular linkage and the composition of its phenolic compounds [44,97]. The effects observed to be hampered, particularly by proanthocyanins, may be associated with tight bonds among fiber and phenolic compounds which result in a delay in degradation and, thus, less fermentation. Nevertheless, Snelders *et al.* [44] observed that fiber fermentability was more related to the amounts of phenolic compounds present in the fiber matrix than to their form (free or bonded to fiber). Thus, performing studies to define the intrinsic composition of dietary phenolics and food fiber could be of great interest for understanding the role of both food components when these are alone or together.

However, it is still questionable whether it is really desirable to stimulate the production of SCFAs. As SCFAs also represent energy extraction of non-absorbed macronutrients, they can provide approximately 10% of the total energy intake [98]. This aspect is considered positive in populations with a diet rich in plant-derived polysaccharide food. Nevertheless, this may be detrimental in Western populations, because a greater capacity to harvest energy from the diet could contribute to the positive calorie intake balance typical of obesity [99]. In fact, the gut microbiota of obese individuals are especially rich in bacteria specialized in carbohydrate fermentation [99,100]. Hindering of SCFAs production observed for some classes of phenolic compounds may be effective in reducing the energy harvest in overweight and obesity, which might partially explain the weight loss observed after the intake of food rich in phenolic compounds [101]. In synthesis, the versatility of phenolic compounds and their association with fiber, free or bound, could stimulate the design of different food products or recommendations depending on the effects expected to be attained.

### 3.2. Bile Acids and Sterols

Bile acids (BAs) are classified into primary and secondary. In humans, the primary BAs cholic and chenodeoxycholic acids are synthesized from cholesterol by hepatic enzymes and stored in the gallbladder (conjugated with the amino acids glycine and taurine) until being excreted into the duodenum to facilitate the digestion and absorption of lipophilic compounds. Most of the conjugated primary BAs are reabsorbed in the ileum and the remaining fraction reaches the colon, where cholic and chenodeoxycholic acids are metabolized into deoxycholic and lithocholic acids, respectively, by gut microbiota [102]. The main microbial catabolism steps include the deconjugation of amino acids and dihydroxylation by microbial 7-α-dehydroxylase [102,103].

Fecal cholesterol represents the non-absorbed dietary cholesterol in addition to that provided by intestinal desquamated cells. The microbial reduction of cholesterol releases coprostanol as the main metabolite, generating cholestanone and coprostanone as intermediates [104]. High excretion of sterols and their microbial metabolites through feces has been associated with hipocholesterolemic effects [105], but is also a common feature of high fat diets [106]. A continuous exposure to elevated luminal levels of secondary BAs and cholesterol microbial metabolites has been suggested to increase the susceptibility to intestinal inflammation and colorectal cancer [74,107,108,109]. In addition to the toxic effects of secondary sterols *per se*, the concentration of putrefactive compounds (see Section 3.3 below) could also increase the lumen toxicity due to the release of amino acids (glycine and taurine), these being products of the microbial hydrolysis of BAs.

There is scarce data regarding the impact of phenolic compounds on the gut microbial transformation of BAs and cholesterol. Different studies have demonstrated that phenolic compounds have suppressing effects on the microbial conversion of sterols. For example, the amount of secondary BAs was reduced in rats fed with a diet containing tea polyphenols and gallotannins [110] or apple, grape, and red beet juices [111]. In another study with rats, the supplementation of a high fat diet with curcumin and caffeic acid reduced the concentration of deoxycholic acid in feces, whereas the administration of caffeic acid, catechin, rutin, and ellagic acid reduced the amount of lithocholic acid [112]. However, there was no evidence of lower conversion of primary to secondary BAs when red wine tannins were added to rat diets [113], or when healthy humans received pomegranate juice over a period of four weeks [85]. Regarding sterols, it seems that dietary phenolic compounds could also influence the conversion of cholesterol into their respective microbial derivatives, especially coprostanol. Compared with a control diet, rats that received tea polyphenols and gallotannins increased their excretion of fecal cholesterol in detriment to the excretion of coprostanol [110]. Similarly, pomegranate juice intake reduces the conversion rate of cholesterol to coprostanol in healthy adults [85]. On the other hand, a large proportion of coprostanol was observed in rats after the intake of apple, grape, and red beet juices [111]. These results suggest that the conversion of primary BAs to secondary BAs and cholesterol to coprostanol is hampered by phenolic-rich diets, but more data regarding the role of phenolic compounds on colonic sterol gut conversion in humans is needed.

The negative implications of the secondary metabolites of sterols in the colon may be mitigated by phenolic luminal compounds. The inclusion of chlorogenic acid in the diet of mice reduced the deoxycholic acid's tumor-promoting effects [109]. In this line, protection against the cytotoxicity effects of deoxycholic acid in Caco-2 cells was observed when cells were incubated with proanthocyanidins [114]. In another *in vitro* study in which deoxycholic acid was incubated with HCT-116 cells, antigenotoxic and aticytotoxic activity was observed for quercetin, resveratrol, and rottlerin [115]. In the same study, genistein, curcumin, and epicallocatechin gallate showed no effect.

High fat diets have also been associated with the increase of the activity of β-glucuronidase [106]. Many toxic metabolites are neutralized by glucuronide conjugation and it is the form that promotes their excretion from the body. Thus, deglucuronidation not only promotes the retention of toxic metabolites in the body but also increases gut environment toxicity. It has been also proposed that phenolic compounds could interfere with the activity of β-glucuronidase, but the results are inconsistent. A decrease in the activity of β-glucuronidase has been observed in rats after diet supplementation with grape extract [92] and ellagitannins in combination with cellulose [94]. On the contrary, higher activity of β-glucuronidase was noticed in rats after the addition of blackcurrant polyphenols to the diet [93].

To date, the effects of phenolic compounds on the microbial transformation of colonic sterols have not been explored in depth and there is no information about the role of phenolic microbial metabolites. Regarding the changes in fecal sterol composition through the effect of phenolic-rich products, the data available in the literature is too scarce to enable solid conclusions to be drawn. Sterols are a non-invasive and easy parameter to be analyzed in fecal samples with a relatively high sensitivity to change when the characteristics of the diet also change [106]. This converts them into good candidates for reflecting alterations in the gut lumen after dietary interventions, particularly those containing high fat.

### 3.3. Products of Non-Absorbed Protein Metabolism

Every day, variable amounts of nitrogenous compounds of dietary and endogenous origins reach the colon. Protein-rich diets, particularly those containing red meat, increase gut toxicity as a consequence of an excessive production of microbial products derived from protein fermentation [64,65,116,117]. These substances, also called putrefactive compounds, include ammonia, fecal phenolic (phenol and *p*-cresol) and indolic compounds (indol and skatole), sulphur compounds (hydrogen sulphide, methyl mercaptan, and dimethyl sulphide), branched chain fatty acids, and polyamides (putrescine, agmatine, cadaverine, tyramine, and histamine). Intestinal ammonia is generated through the microbial deamination of urea and amino acids [65,118]. Phenolic and indolic compounds are mainly derived from the microbial catabolism of aromatic amino acids (phenylalanine, tyrosine, and tryptophan) [65]. Sulphate-reducing bacteria produce sulphur metabolites as a product of anaerobic respiration during the catabolism of cysteine and methionine [119]. Polyamides, besides being present in food, are also released in the gut by the bacterial metabolism [120].

Elevated undigested luminal proteins could alter the intestinal ecology, stimulating the activity of nitrogen-degrading bacteria, which are considered to have detrimental effects in gut homeostasis [121,122]. In parallel, microbial enzymatic expression and activity increase proportionally with the amount of substrate [122], thus promoting the accumulation of putrefactive compounds which are associated with gut integrity and function loss due to their potential cytotoxicity, genotoxicity, and carcinogenic activity, increasing the risk of inflammatory bowel disease (IBD) and the development of colorectal cancer [66,74,116,123].

The modification of dietary patterns, such as including dietary carbohydrates [89,116,117], and restricting energy intake [124], has shown an inverse relationship with the concentration of these putrefactive fecal compounds. However, there is insufficient information regarding the effects of phenolic compounds in this aspect. Among the limited number of studies focused on researching the relation between protein fermentation and phenolic compounds, the most numerous are those on polymeric and monomeric flavan-3-ols. In the case of animal studies, a tea phenol-enriched diet reduced the pH and concentration of ammonia, phenol, *p*-cresol, and skatole in pig feces after two weeks of treatment [81]. Satisfactory results were also observed in humans after supplementation with grape seed extract [125] and flavan-3-ols [126]. Both products reduced fecal pH and hampered the production of sulphur compounds. A trend toward a decrease in phenol, *p*-cresol, 4-ethylphenol, indole, and skatole in feces was observed after consumption of grape seed extract, but these compounds were not studied after flavan-3-ol supplementation. In the case of ammonia, its fecal amounts were significantly reduced in the flavan-3-ol group and tended to decrease after grape seed extract intake. More consistent results, observed after the intervention with flavan-3-ols, may be related to the duration of the study, this being two and six weeks for grape seed extract and flavan-3-ols treatments, respectively. On the contrary, no changes in the production of ammonia and sulphides were noticed after continuous feeding of soygerm powder in a SHIME system [61].

A reduction of the pH of the feces observed after phenolic intervention could partly explain the decrease in the concentration of putrefactive substances, since microbial proteases are more active at neutral or slightly alkaline pH [127]. However, this is not a common effect. In some cases, phenolic compounds increase [92] or have no effects [80,83] on lumen pH. In addition, a reduction in fecal putrefactive compounds does not always respond to a reduction in the pH [80,92]. The differences may depend on the predominant class of bacteria and the availability preference of the substrate type. For example, *Escherichia coli* and *Proteus mirabilis* produced ammonia from amino acids even at low pH [118].

Phenolic compounds and their microbial metabolites possibly collaborate with additional factors to reduce the accumulation of protein fermentation products. A reduction of protein conversion, beyond the decrease in pH in the gut lumen, may also be associated with the capacity of phenolic compounds to inhibit proteolytic bacteria and interfere with their enzymatic activity. Gram-negative Proteobacteria (especially *E. coli*), *Bacillus* spp., and Gram-positive Clostridia [118,128] have been implicated in gut proteolysis and their abundance in IBDs is probably associated with the typical morphological and physiological alterations [129,130]. Beside the capacity of phenolic compounds to inhibit the growth of several members of the proteolytic bacteria, it has been observed that phenols also possess the capacity to attenuate the expression of genes involved in the secretion of proteases, inhibiting their synthesis and their activity [125,131,132,133]. The degree to which the enzymatic activity is blocked depends on the phenolic concentration, its molecular structure, and its functional groups [133].

Abnormal concentrations of proteinases and putrefactive compounds in the lumen alter the permeability of the gut epithelium, promoting the movement of toxic substances across the intestinal barrier, which leads to a tendency to activate inflammatory mediators [129,134]. One of the reported mechanisms by which phenolic compounds improve barrier integrity is by increasing the expression of tight junction proteins [84,134,135,136,137]. This may mean that although some phenolic compounds are unable to reduce protein fermentation, they offer an alternative way of protecting colon integrity, enhancing the host tolerance to susceptible molecules present in the internal environment.

Large intestine digestion is a dynamic system in which entrapped compounds in the digesta pass through the ascending, transversal, and descending colon before being excreted by feces. Local microbiota is active throughout the lumen, metabolizing non-absorbed products in the function of substrate preference and availability, and is also conditioned by the physiological conditions of the gut (pH, redox potential, transit time) [138]. The pattern of the native phenolic compounds present in food change between the colonic segments due to the microbial fermentation, resulting in the accumulation of different metabolites with different activity than their precursors. As major proteolytic activity is found in the terminal colon [64,123], it would be interesting to study the interaction of microbial proteolysis-phenolic metabolites in this part of the gut. In addition, we have found only one paper related to the study of the modification of fecal nitrogen compounds after the addition of phenolic-rich products in a high red meat diet in humans [139]. However, to have a better understanding of the role of dietary phenolic compounds in protein fermentation at gut level, more human and animal studies are needed.

## 4. Impact of Phenolic Compounds on Intestinal Function

### 4.1. Intestinal Redox Homeostasis

Oxidative stress is defined as the damage promoted by an imbalance between pro-oxidant agents (free radicals) and endogenous (superoxide dismutases, SOD, catalase glutathione peroxidase, GPx, and glutathione, GSH) and exogenous mechanisms to neutralize their effects. Free radicals are highly reactive molecules capable of reacting with the biological components of cellular structures, such as the structural lipids of cell membranes, proteins, and nucleic acids (DNA damage), compromising good cell function and integrity. Moreover, the alteration of cellular components caused by oxidative stress in many cases precedes the over-expression of pro-inflammatory agents that are responsible for the activation of mechanisms that trigger cell damage [140]. Repeated bouts of inflammation can lead to the occurrence of IBDs, such as ulcerative colitis and Crohn’s disease, which are, in turn, risk factors of colon cancer [141].

Pro-oxidant substances are products of normal aerobic metabolism, but their levels increase as a consequence of toxic exposure (smoke, excessive alcohol intake, irradiation, some drugs, food components, toxins) and other particular situations (age, infection, stress). The gastrointestinal tract produces, receives, and retains these types of substances, which implies an elevated risk of alterations in cells and tissue in situations of over-production of free radicals and/or depletion of antioxidant reparative mechanisms [142]. Dietary phenolic compounds could act as an antioxidant system in the gastrointestinal lumen and epithelium, reducing the oxidative damage in different ways: safeguarding the activity of endogenous antioxidants, decreasing free radical production, or neutralizing the latter by radical scavenging [6,7,9,140,143,144,145,146,147].

*In vitro* studies using cell-based methods are considered a good tool for deciphering the mechanisms by which phenolic compounds attenuate oxidative responses. Treatment of intestinal Caco-2 cells with different fractions of cranberry and apple peel phenolic extracts [140,143], as well as red wine extracts [144], effectively mitigated the membrane structure disruption caused by the pro-oxidants Fe/ascorbate and tertra-butyl hydroperoxide, respectively. Direct scavenging activity is the proposal mechanism by which urolithins suppress the generation of free radicals from activated neutrophils [145]. On some occasions, foods also collaborate with the pro-oxidant potential of the gut environment. For example, an overproduction of free radicals and a reduction of GSH activity were evident in Caco-2 cells when they were incubated with acrylamide, but the latter effects were reduced by cocoa phenolic extract, procyanidin dimer B2, and epicatechin [146]. In a similar way, protection against oxidative damage induced by the micotoxin deoxynivaleol in HT-29 cells was also observed when the culture media was enriched with epigallocatechin gallate [147].

The main entrance route for phenolic compounds is the gastrointestinal tract, where they reach a greater lumen concentration and remain longer compared with those observed in blood circulation [22,62]. Also, the extensive phase II metabolism (glucuronidation, sulphatation, and methylation) that phenolic compounds undergo during absorption and body distribution created doubts concerning their effectiveness as antioxidant agents at the systemic level [13,145]. In the case of colon lumen, the non-absorbed phenolic compounds and their microbial metabolites prevail in native-unconjugated forms [148]. During digestion, the antioxidant capacity of the luminal content can indeed increase compared with the original food, probably due to the release of phenolic compounds from the food matrix promoted by the physiological conditions and action of digestive enzymes [149]. In the large intestine, microorganisms have the ability to release those phenolic compounds intimately entrapped in the food matrix (particularly dietary fiber), which also contribute to the total antioxidant capacity at the gut lumen level [150,151]. Moreover, the phenolic compounds released from the digestive bolus can be distributed into intestinal fluids and favor direct contact with the colonic epithelium, mitigating the detrimental effects of free radicals at the cell level.

The antioxidant status in *in vivo* studies (animals or humans) could be estimated by analyzing the antioxidant capacity of the intestinal content or fecal water by different methods, such as the oxygen radical absorbing capacity (ORAC), the ferric reducing antioxidant power (FRAP), the diphenyl-1-picrylhydrazyl (DPPH), the free radical scavenger (TBARS), and the 2,2′-azinobis-(3-ethylbenzothiazoline-6-sulphonate) radical cation (ABTS) [6,13,14,19,20,149,150,151]. The total antioxidant capacity of the intestinal content increased after the ingestion of grape seed extract in rats [151] and limited the production of free radicals in human fecal water after polyphenol-rich chocolate intake [152]. In contrast, some studies have noted no changes in the antioxidant capacity in colon tissue after the intake of phenolic-rich products [10]. The difference in the bioactivity between microbial phenolic metabolites and their parent compounds together with the progressive decrease of the lumen phenolic concentration due to the active colonic absorption could be an explanation for the unobserved antioxidant effects. However, it should be stressed that lack of effect in the total antioxidant capacity does not necessarily encompass the loss of other biological properties [10] (see next Section 4.2.).

### 4.2. Intestinal Inflammation

Inflammation is a biological phenomenon associated with the innate body immune response in the first line of defense to combat threats which could compromise human health. Episodes of controlled inflammation are indispensable for suppressing chemical, biological, and physical injuries. However, in some cases, due to diverse genetic and environmental causes, uncontrolled inflammation responses predispose individuals to the development of chronic diseases. In the case of the gastrointestinal tract, IBDs are characterized by an unknown etymology of recurrent episodes of inflammation causing a progressive loss of cellular function and tissue degeneration that could develop into colon cancer [141,153].

The chronification of inflammation involves complex and interconnected mechanisms of molecular mediators, whose continued feedback promotes the perpetuation of inflammation. The over-production of free radicals (reactive oxygen and nitrogen species) and cytokines (TNF-α, IL-6, IL-8, PGE_2_, among the most studied), the up-regulation of nuclear factor kappa-B (Nf-κB) and monophosphate-activated protein kinase (MAPK), and the recruitment, activation, and adhesion of leukocytes have been identified as occurring in IBDs [7,8]. Phenolic compounds seem to inhibit or attenuate the intensity of the inflammatory response by modulating the cellular inflammatory mediators and/or neutralizing free radicals (see Section 4.1 above).

*In vitro* cultures of intestinal cells are particularly valuable models for identifying the target molecules involved in chronic inflammation which may be susceptible to regulation by phenolic compounds. The results obtained from these studies could be interesting for the design of new anti-inflammatory drugs capable of avoiding the adverse side effects observed for some drugs used to treat IBDs [154]. The possible mechanisms by which phenolic compounds could mitigate or suppress inflammatory responses *in vivo* can be predicted by cells activated by different types of inflammation promoters. The down-regulation of NF-κB is considered a therapeutic target in IBDs, since it prevents the activation of pro-inflammatory mediators, such as COX-2 and iNOS, which, in turn, are responsible for the production of cytokines and free radicals. Individual compounds, such as epigallocatechin gallate [147] and anthocyanins [155], and also phenolic mixtures obtained from apple peels [143], cocoa [8], cranberries [140], and red wine [156], have shown an *in vitro* ability to inhibit the activation of NF-κB pathways. In the latter studies, the inhibition of NF-κB activity was also associated with the down-expression of COX-2 [8,143,147,156] and iNOS [8,140,156]. Resveratrol [157] and cocoa phenolic compounds [146] were also effective as regulators of other signaling pathways such as JAK-STAT and MAPK, respectively. On the contrary, chlorogenic-rich fractions obtained from lyophilized blueberries did not show clear evidence as inhibitor agent of NF-κB [155].

Although less common compared with the data available regarding the anti-inflammatory potential of phenolic compounds, it seems that their microbial metabolites also possess similar properties. The down-regulation of COX-2 in human adenoma cells was observed after the incubation of (3′,4′-dihydroxyphenyl)acetic and 3-(3′,4′-dihydroxyphenyl)propionic acids [158], common phenolic catabolites detected in feces. Reclusion and adhesion of leukocytes play an important role in the development of inflammation since they also secrete pro-inflammatory cytokines. The secretion of cytokines TNF-α and IL-6 was effectively inhibited by urolithins after activation of THP-1 human monocyte cells [159]. Urolithins were also effective as anti-inflammatory mediators in activated neutrophils, inhibiting cytokines and the secretion of the proteinases necessary for adhesion [145]. Fibroblasts are a type of cell that synthesizes the extracellular matrix and collagen, responsible for tissue cicatrization. Their activation, mediated by cytokines, monocytes, and free radicals, also involves the secretion of pro-inflammatory agents. In addition, the exacerbated extracellular production of collagen could lead to intestinal stenosis (bowel obstruction) [160]. Urolithins, especially urolithin A, were also effective at inhibiting the activation, migration, and adhesion of colon fibroblasts with a concomitant reduction of several secretory pro-inflammatory mediators. These effects were even more evident than those shown by their precursor, ellagic acid [161].

Chemically induced IBD in animals is a revolutionary approach to studying the effect of phenolic compounds on inflammation under *in vivo* conditions [153]. The improvement in physical indicators (less body weight loss) and hematological and histological probes, together with a reduction in the expression of pro-inflammatory mediators in combination with phenolic treatment, constitute evidence of the protection given by phenolic compounds against intestinal epithelium damage and the attenuation of the inflammatory response. Individual phenolic compounds [7,10,134,136,162], and phenolic extracts [7,8,163,164] have shown positive effects in the control of IBDs in animals, even to the same extent as sulphosalizine, a common medicine used in IBDs, but with fewer collateral effects [7]. Contrary to the later results, the administration of resveratrol to rats for six weeks induces the expression of inflammatory parameters [84]. Data from animal studies support the anti-inflammatory effect of phenolic compounds observed in *in vitro* experiments, but the role of phenolic metabolites in this aspect is still unclear, due to the lack of information about the phenolic disposition in tissues and feces in most of the later studies.

Dietary intervention in human studies including IBD patients is basically conducted to observe the remission of clinical symptoms or the diminution in the number or intensity of relapses [165,166,167]. Despite phenolic compounds have been extensively studied in animal models of IBDs, data regarding human trials is currently limited to one study in which the administration of epigallocatechin gallate to patients with ulcerative colitis over 56 days showed a satisfactory degree of remission of symptoms with minimal side effects [166]. Due to the ethical issues mentioned above, human studies to investigate the *in situ* effects promoted by phenolic compounds are not always possible. Nevertheless, there are promising fecal inflammatory markers that could potentially be used in human studies with minimal invasiveness.

Calprotectin and lactoferrin are binding proteins found in the cytoplasm of neutrophils, monocytes, and macrophages. Fecal calprotectin could be a good tool for the molecular screening of active intestinal inflammation, because its levels are substantially elevated in IBDs and colorectal cancer [167,168,169]. Furthermore, the amounts of calprotectin in feces have been shown to correlate well with the histological lesions caused by continuous inflammation in IBDs [167,168,170] and the tumor stage in colorectal cancer [169], indeed with better results than other common plasma biomarkers, such as the case of C-reactive protein [167,170]. A reduction in the fecal excretion of calprotectin was observed after surgical intervention [169] and oral probiotic administration [171]. Rats fed with a high fat diet in combination with grape seed extract showed lower levels of fecal calprotectin than the control animals [172].

Although collecting feces is easy and non-invasive, human studies concerning fecal inflammation biomarkers are scarce. To our knowledge, there is only one study involving IBDs patients and phenolic treatment. In this work, anthocyanin-rich bilberries were administrated to ulcerative colitis patients for nine weeks and the results at the end of the study showed a decrease in the fecal levels of calprotectin, which increased again when the bilberry treatment was stopped [173]. These results not only show the palliative effects of some phenolic compounds in IBDs, but also the inclusion of calprotectin as an index of inflammation response after dietary treatment. Exploring the association between phenolic compounds and intestinal inflammation through dietary treatment is a promising future approach to be considered.

## 5. Phenolic Compounds and Gut Microbiota Modulation

The gastrointestinal tract, especially the large intestine, houses the most abundant and complex microbiota in humans. The gut population, composed of approximately 10^10^–10^12^ cells per gram of intestinal content, participates in several metabolic functions that the host cannot fulfill by itself [174,175,176]. These metabolic functions and their derivative end products depend on the quantitative and qualitative features of the gut inhabitants. A harmonious balance in the composition of the gut microbiota has been associated with maintaining health and a higher life expectancy accompanied by a satisfactory quality of life. An imbalance in the microbial population is known as dysbiosis. Dysbiosis has been associated with intestinal and non-intestinal metabolic disorders, which confirms the association between the microbiota and non-digestive functions [176].

Although full characterization of the microbiota in homeostasis and disease still remains to be completed, some trends in its profile could be helpful for discriminating between the two states. A lower diversity of bacteria, change in bacteria functionality, and a decrease in the beneficial inhabitants together with a major abundance of detrimental opportunistic bacteria seem to be involved in metabolic alterations such as obesity and type-2 diabetes, and diseases of the gut such as IBDs and colorectal cancer [9,10,73,100,177,178]. An alteration of the microbial equilibrium undermines bowel functionality, alters host immunity, and increases susceptibility to pathogen colonization.

The mechanisms by which the phenolic compounds modulate the gut microbiota still remain to be deciphered, but may involve direct and indirect interactions. Phenolic compounds could directly stimulate or inhibit bacterial growth. Inhibition is closely related to previously reported antimicrobial properties of phenolic compounds and stimulation presumably associated with the capacity of the bacteria to metabolize them [131,179]. The antimicrobial activity of phytochemicals has been extensively studied because is thought that it is the main cause by which phenolic compounds can modify the characteristics of the gut structure population. On the basis of several works [131], it could be said that phenolic compounds possess a selectively bacteriostatic or bactericide effect, inhibiting the growth of a wide range of potential pathogenic bacteria slightly affecting or even promoting the beneficial microbial population. However, it is important to consider the concentration and characteristics of the molecule (type of phenolic compound and if it is presented in conjugated or free form) because these factors seem to govern the modulation of the desired effects. Indirect implications include complex issues where numerous microbial-host and microbial-microbial interrelationships take place. Phenolic metabolites could affect the growth of other bacterial groups and, in turn, the over-growth of some bacterial groups could condition the development of others [76,179,180].

The prevalence of determinate microbiota members is preferred to others due to the efficacy they have shown in ameliorating the gut ecosystem with positive effects at the local and systemic levels. For this reason, most studies have focused on the effects of polyphenols on *Bifidobacterium* and *Lactobacillus*, but there is increasing interest in other emergent bacteria that could be relevant for the health of the host [9,101]. Under *in vitro* conditions, anthocyanins [38], phenolic compounds bound to the insoluble cocoa fraction [150], pomegranate extract [45], and soygerm powder [61] have stimulated the growth of *Lactobacillus* and *Bifidobacterium*. Pomegranate extract and apple pomace juice were also capable of increasing *Bifidobacterium* levels in rodents [111,163]. The *Lactobacillus* count was increased after the administration of apple and red beet pomace juice instead of water in rats [110]. Grape seed extract rich in proanthocyanins was able to increase the population of *Bifidobacterium* in healthy adults [125]. Although the phenolic compounds contained in a cocoa powder did not prevent the age-induced reduction of *Lactobacillus* and *Bifidobacterium* in rats after six weeks, they reduced the growth of Bacteroidetes, *Staphylococcus*, and *Clostridium* [181]. The latter prebiotic effects could also have been related to the possible presence of fiber in the phenolic sources. Dietary fiber is known to influence the microbiota profile, especially through the increase in probiotic microorganisms [182]. However, other sources deprived of dietary fiber, as in the case of incubation of individual compounds [76], green tea phenolic compounds in pigs [81], fiber-free juice [101], resveratrol administrated to rats [10], isoflavone supplementation in women [183], and cocoa-isolated phenolic compounds in healthy adults [184], have also shown positive effects to favor the increase in the number of *Bifidobacterium* [10,76,183,184], *Lactobacillus* [10,101,184], and butyrate-producing bacteria in parallel with the modulation of growth of bacteria associated with gut dysbiosis [10,76,184].

Diet is probably the most easily manipulable factor to modulate the composition of the gut microbiota, and the latter results show that phenolic compounds *per se* are presumably able to interact with gut inhabitants and produce changes, most of them toward a healthier profile. However, in a recent study by our group [85], no changes were observed in the microbiota composition of healthy adults after four weeks of sustained intake of pomegranate juice with a high phenolic content. Therefore, the production of the microbial phenolic metabolites catechol and phenylpropionic acid was correlated with the presence of a higher percentage of some bacterial groups, supporting the existence of phenolic compound-bacteria interrelationships. Despite this fact, the moderate consumption of red wine by healthy volunteers over a period of 20 days increased the number of Firmicutes, Bacteroidetes, *Bifidubacterium*, and *Prevotella* [185]. The *Bifidobacterium* population also increased in the feces of healthy humans after six weeks of sustained intake of blueberry drink [186], basically promoted by the higher amounts of some species [187].

Recent studies have proposed the use of phenolic-rich sources as a therapeutic strategy to prevent and, in some cases, reverse the dysbiosis associated with different pathologies. Obese rodents or animals fed with obesogenic diets are common tools for studying the role of the microbiota in the metabolic syndrome. The metabolic syndrome includes a series of alterations, such as obesity, dyslipidaemia, glycosemia, and loss of insulin sensitivity. Thus, the attenuation of one of the latter factors could be considered as a protective factor of type 2 diabetes and cardiovascular diseases. A high Firmicutes/Bacteroidetes ratio has been considered an index of obesity and its depletion a marker of successful treatment. However, the results in this context are ambiguous. The reduction in the incidence of obesity reflected by lesser weight gain and fat accumulation was observed after treatment with quercetin plus resveratrol [84], coffee [188], fiber-free plum [101] and cranberry extract [9], but no significant differences were observed after pomegranate peel extract intake by obese mice [163]. The majority of the latter phenolic treatments showed a modification of the microbial population with respect to their control counterparts, but it was not always encompassed by a decrease in the Firmicutes/Bacteroidetes ratio, suggesting the involvement of other bacteria or non-microbiota mediated effects. Nevertheless, a positive correlation between body mass index and the Firmicutes/Bacteroidetes ratio is still questionable because this parameter has not always been described in obesity [99,101,189]. The prevention of obesity is a target factor to reduce the incidence of type 2 diabetes, which could be reinforced by the additional effects provided by dietary phenolic compounds. For example, the administration of cranberry extract to mice for nine weeks increased the relative abundance of *Akkermansia* ssp., which has been linked with the enhancement of diabetic protective effects such as an increase in insulin sensitivity and better inflammatory parameters [9].

IBDs have also been associated with a microbial status in which *Enterobacteriacea*, especially *E. coli*, are more abundant than in the control subjects. The characterization of *E. coli* has revealed that the virulent types were more abundant in biopsy specimens in IBDs patients compared with healthy individuals, basically due to the expression of adhesines which facilitate the adherence of the bacteria to the epithelium surface [190]. The administration of resveratrol to IBD-induced rats for 25 days prevented the over-growth of *Enterobacteriaceae*, particularly *E. coli* [10]. Some studies confer the phenolic compounds with the *in vitro* properties of inhibiting the cellular adhesion of harmful bacteria with a minimal repercussion on the beneficial members of the gut microbiota [191].

All seem to indicate that the gut community responds positively to dietary phenolic compounds where the abundance of determinate bacteria could contribute to host health maintenance. After phenolic intervention, microbiota changes may by expressed on different taxonomic levels. Lack of difference at the phylum level does not necessarily indicate a lack of probiotic effects; sometimes the changes are evident at class, family, genus, or species levels [84].

Microbiota profile and, thus, microbiota modulation after diet intervention can be studied by different techniques, such as fluorescent *in situ* hybridization (FISH), quantitative PCR-based methods (qPCR) [76,101], and high-throughput sequencing; these are among the most widely used. The analyses by FISH and qPCR are based on identifying and quantifying specific bacterial groups, defined as oligonucleotide probes. For this, in most of the assays, the selected probes depend on the specific bacteria of interest in the study and probably some important microbial changes may be omitted if the probe is not considered in the study. Other, more sophisticated techniques are based on the amplification and pyrosequencing of bacterial 16S ribosomal RNA and allow overall population bacterial genoma identification (metagenome) [101,177]. A more in-depth and detailed characterization of the whole microbiome provides information regarding changes not only in the phylum, class, family, and genus but also specific alterations observed at the species level. In summary, the phenolic compounds that may hamper the microbial imbalance in situations of risk of disease or those that may prevent dysbiosis by reinforcing beneficial bacteria could be included in therapeutic strategies to restore or maintain a healthy profile of the gut microbiota. It remains to be elucidated whether dysbiosis is a cause or consequence of several human pathologies associated with the microbiota.

## 6. Conclusions and Future Perspectives

The large intestine is a sophisticated and complex ecosystem where interrelationships among the host, microbiota, and its metabolic products (dietary and endogenous products) play a key role in local and systemic health. The inter-dependence between the latter factors is so tight that the alteration of one of them can govern the behavior of the others, altering the gut homeostasis and, thus, making the appearance of disease more likely.

Dietary phenolic compounds reach the colon in variable amounts, enriching the gut lumen of the related metabolites derived from the local microbiota metabolism. Lumen phenolics (parent compounds and their metabolites) can actively take part in the innumerable interactions that occur in the large intestine. Depending on their nature, diet phenolic compounds apparently enhance the generation of beneficial metabolic products and/or hamper the production and effects of detrimental luminal compounds, protecting and prolonging gut homeostasis. The mechanisms by which phenolic compounds can interact with the gut metabolism may involve interference with enzymatic expression and activity, changes in the characteristics of the gut environment, changes in the modulation of the signaling pathways responsible for pro-oxidant, inflammatory, and carcinogenic effects, enhancement in the protection of the intestinal epithelium from the negative effects of toxic substances, and/or the modulation of the microbial population.

There are still several aspects that require emphasizing. Phenolic compounds undergo active colonic microbial transformations, generating intermediate and final related metabolites that could be present in the digesta in higher concentrations than their precursors, without necessarily sharing the same bioactivity. In this context, studies focused on analyzing the parent compounds in food probably fail to estimate the phenomena occurring in the colon correctly. It would be important to identify whether the health benefits are associated with the parent compounds or their respective microbial derivatives in order to design the correct dietetic or technological strategies to obtain major and better results from phenolic dietary therapies.

Phenolic compounds represent a wide group including several sub-groups that, although they have similar characteristics, are not completely equal. Differences in chemical structure, number of functional groups, and the combination of different moieties probably lead to these molecules having different functions. Despite the information obtained and published over recent years, the effects and mechanisms by which phenolic compounds promote changes in the human gut ecosystem are not yet completely elucidated. A solution could be partly promoted by the combination of metabolomic (characterization of metabolite changes in the gut environment after dietary intervention with phenolic compounds or phenol-rich foods), microbiome (gut bacteria collective genome), and metagenomic (for the identification of down- or over-expressed proteins involved in microbial activities, whose classification thus depends on the function) disciplines to describe a more complete scenario promoted by phenolic compounds.

Given all of the above, the design and performance of more research, especially focused on human trials, is encouraged to confirm the efficacy of phenolic compounds at the gut level. The results provided by previous and future studies could be useful to the design of dietary recommendations not only to suppress or reduce symptoms in disease but also to provide the healthy population with simple tools to promote the maintenance of health.
